# Phosphodiesterase signaling in asthma: mechanistic insights and emerging therapeutic strategies

**DOI:** 10.1007/s00210-026-05098-y

**Published:** 2026-02-17

**Authors:** Abdulelah Alnusayri, Hayder M. Al-kuraishy, Athanasios Alexiou, Marios Papadakis, Safaa A. Faheem, Mubarak Alruwaili, Gaber El-Saber Batiha

**Affiliations:** 1 College of Medicine, Jouf University, Sakaka, Saudi Arabia; 2 Department of Clinical Pharmacology and Medicine, College of Medicine, Al-Mustansiriya University, Baghdad, Iraq; 3 European Academy of Sciences and Arts, Vienna, Austria; 4 University Centre for Research & Development, Chandigarh University, Chandigarh-Ludhiana Highway, Mohali, Punjab India; 5 Medical Department, Faculty of Life Sciences, University of Thessaly, 3 Panepistimiou Str., Viopolis, Larissa, 41500 Greece; 6 Department of Pharmacology and Toxicology, Faculty of Pharmacy, Egyptian Russian University, Cairo-Suez Road, Badr City, Cairo, 11829 Egypt; 7 Department of Internal Medicine, College of Medicine, Jouf University, Sakaka, Saudi Arabia; 8 Department of Pharmacology and Therapeutics, Faculty of Veterinary Medicine, Damanhur University, Damanhur , AlBeheira, 22511 Egypt

**Keywords:** Asthma, Phosphodiesterases, Phosphodiesterase inhibitors, Airway smooth muscle

## Abstract

Asthma is a clinically and biologically heterogeneous disease for which current anti-inflammatory therapies, including inhaled corticosteroids, remain insufficient for many patients. Therefore, repurposing other FDA-approved drugs, such as phosphodiesterase (PDE) inhibitors, may be beneficial in the management of asthma. PDEs are central regulators of cyclic nucleotide signaling, modulating airway smooth-muscle tone, mucociliary function, and immune-cell activity by compartmentalizing the control of cAMP and cGMP. Although PDE4 inhibitors have shown strong pre-clinical efficacy, their translation into clinical practice has been hindered by dose-limiting systemic toxicities, revealing a persistent gap between mechanistic promise and therapeutic utility. This review synthesizes emerging insights into PDE isoenzyme selectivity, spatial localization, and cross-regulatory interactions across structural and immunocompetent airway cells. We outline how isoform- and splice-variant–specific functions contribute to key asthmatic phenotypes and evaluate innovative therapeutic strategies, including dual PDE3/4 inhibition, inhaled delivery approaches, and next-generation selective modulators designed to overcome historical limitations. By integrating these advances within an endotype-driven precision-medicine framework, this review provides a path toward unlocking the full therapeutic potential of PDE-targeted interventions in asthma.

## Introduction

Asthma is a reversible airflow obstruction caused by airway hyperresponsiveness and airway inflammation (Rackemann 1947). For decades, all individuals with airway lability and fluctuating chest symptoms have been given the uniform label of having “asthma,” yet there is little doubt that there is significant heterogeneity among those who suffer. Asthma “phenotypes” have been described in the literature since the early 1940s. Rackemann (Rackemann 1947). Originally described the phenotypes of “intrinsic” vs “extrinsic” asthma. In that report, Rackemann noted that while patients with “intrinsic” asthma commonly developed asthma after age 40 and tended to have less sensitization despite relatively low lung function, patients with “extrinsic” asthma developed asthma in childhood in concert with allergic sensitization. Two groups of patients with severe, systemically corticosteroid-dependent asthma were distinguished, based on the presence or absence of airway eosinophils, about 50 years later (Wenzel et al. 1999). Even in the absence of eosinophils, there was less evidence of airway remodeling; these patients nonetheless had significant symptoms and an overall somewhat reduced forced expiratory volume in one second (FEV1) despite receiving large amounts of anti-inflammatory therapy (Wenzel et al. 1999).

**Fig. 1:**
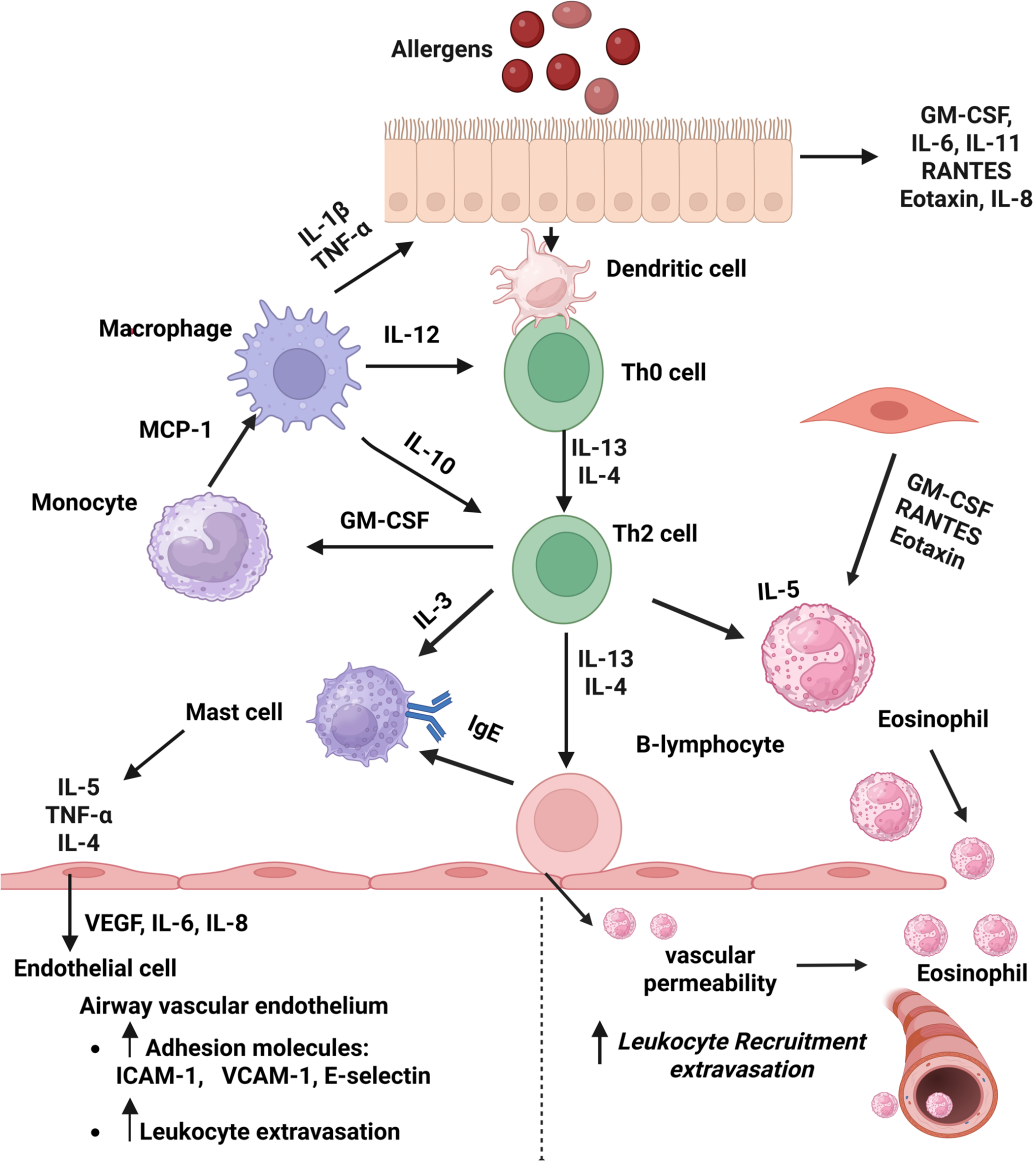
Schematic representation of the inflammatory cascade in allergic asthma involving immune and structural airway cells. Following allergen exposure, airway epithelial cells release pro-inflammatory mediators and chemokines that activate antigen-presenting dendritic cells, leading to Th0 polarization toward a Th2 phenotype. Th2-derived cytokines (IL-4 and IL-13) promote B-lymphocyte class switching and IgE production, resulting in mast-cell activation, while IL-5 supports eosinophil maturation and recruitment. Monocyte–macrophage signaling further amplifies airway inflammation through cytokine release. Airway vascular endothelial cells contribute to asthma pathogenesis by increasing vascular permeability and edema and by upregulating adhesion molecules (ICAM-1, VCAM-1, and E-selectin), thereby promoting leukocyte recruitment and extravasation into the airway wall. Abbreviations: GM-CSF, granulocyte–macrophage colony-stimulating factor; IL, interleukin; MCP-1, monocyte chemoattractant protein-1; RANTES, regulated upon activation, normal T-cell expressed and secreted; Th, T-helper; TNF-α, tumor necrosis factor-alpha

Of note, many factors influence asthma prevalence, including sex, race and ethnicity, poverty level, nation location, and Metropolitan Statistical Area (a measure of urbanicity). In particular, asthma prevalence was highest from 2001 to 2010, before marginally declining between 2010 and 2017 (Su et al. 2019). A broad spectrum of molecular pathways, inflammatory cells, and soluble mediators contribute differently to the diverse clinical phenotypes that characterize asthma’s complex etiology (Wenzel 2004). Although many immune cell populations participate in the disease process, eosinophils have traditionally been viewed as a hallmark feature of the most common histopathological pattern. Their precise role, however, remains debated. Following allergen exposure, plasma cells derived from memory B lymphocytes produce IgE, which subsequently triggers mast cells and basophils to release multiple inflammatory mediators, including interleukins 1 through 5, granulocyte–macrophage colony-stimulating factor (GM-CSF), interferon-γ (IFN-γ), and tumor necrosis factor-α (TNF-α) (O’Byrne et al. 2001). These mediators promote bronchoconstriction and recruit additional inflammatory cells, including neutrophils, T lymphocytes, and macrophages. Interleukin-5 is particularly notable because it governs the maturation and mobilization of eosinophils from the bone marrow. Additional chemokines, including eotaxin, macrophage inflammatory protein-1α, and RANTES, secreted upon activation in normal T cells, facilitate the trafficking of these immune cells into the airway (O’Byrne et al. 2001). Indeed, monoclonal antibodies targeting interleukin-5 lead to marked reductions in eosinophil counts in tissue and sputum samples but only minimal effects on bronchial hyperresponsiveness or overall disease trajectory in many patients (O’Byrne et al. 2001) (Fig. 1).

Furthermore, phosphodiesterases (PDEs) have been implicated in the pathogenesis of asthma, and PDE4 inhibitors are effective in reducing asthma severity (Al-Sajee et al. 2019). For example, the PDE4 inhibitor roflumilast has been shown to diminish the late-phase asthmatic response and prevent the associated increase in airway reactivity after allergen exposure. However, it exerts little influence on the immediate bronchoconstrictive phase (Kapui et al. 1999). Furthermore, when used as an adjunct to inhaled corticosteroids or montelukast, roflumilast has demonstrated additive, sustained improvements in lung function in asthmatic patients (Bodkhe et al. 2020). Several other PDE4 inhibitors, including cilomilast, revamilast, MEM 1414, and BLX-028914, progressed into clinical development for asthma but were ultimately discontinued (Matera et al. 2014). Consequently, no PDE4 inhibitor has been approved for the treatment of asthma. Still, the systematic review and meta-analysis of major databases from 1946 to 2016 have found that oral PDE4 inhibitors may serve as an alternative to standard bronchodilators and inhaled controllers in patients with mild asthma (Luo et al. 2018).

Thus, this narrative review aims to provide a critical synthesis of the complex role that PDEs play in the pathophysiology of asthma. The key rationale lies in bridging the translational gap between the clinical failures of systemic PDE4 inhibition and the strong preclinical promise of PDE modulation. In this context, this review has two objectives: (1) to review the molecular underpinnings of PDE isoenzyme function and compartmentalization in key lung cells; and (2) to assess how dual-PDE inhibition can be utilized within a precision medicine framework to develop PDE-targeted therapies for asthma.

## An overview of PDEs

**Fig. 2:**
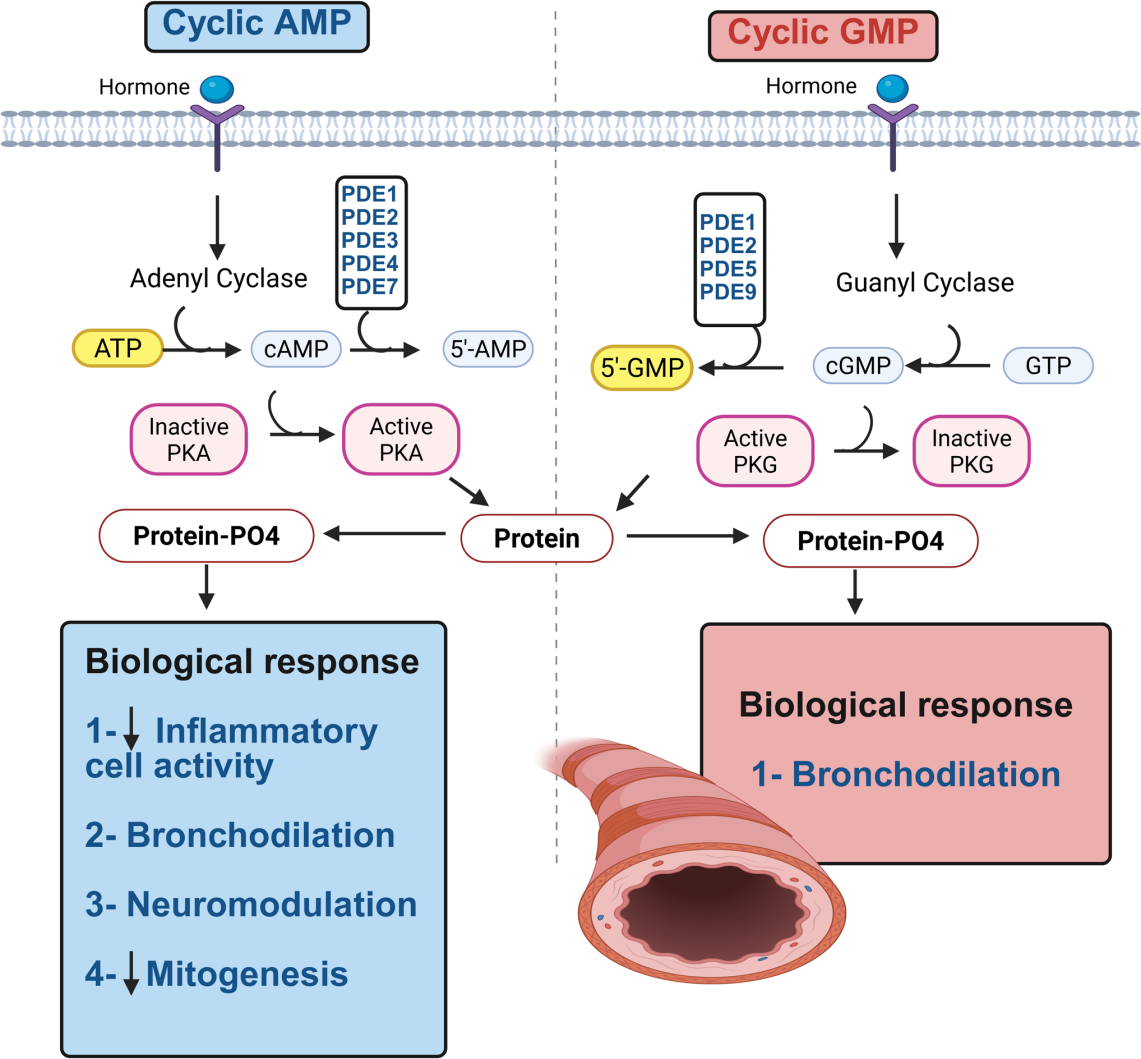
Overview of cyclic nucleotide signaling pathways and phosphodiesterase (PDE)-mediated regulation. Extracellular stimuli activate adenylyl cyclase or guanylyl cyclase, respectively, to generate cyclic AMP (cAMP) from ATP or cyclic GMP (cGMP) from GTP. cAMP activates protein kinase A (PKA), whereas cGMP activates protein kinase G (PKG), leading to downstream phosphorylation of target proteins and subsequent biological responses. PDEs terminate cyclic nucleotide signaling by hydrolyzing cAMP to 5′-AMP or cGMP to 5′-GMP. PDE isoenzymes differ in substrate specificity, with some preferentially hydrolyzing cAMP (e.g., PDE3/4/7), others preferentially hydrolyzing cGMP (e.g., PDE5/9), and certain families showing dual-substrate activity (e.g., PDE1/2). Abbreviations: cAMP, cyclic adenosine monophosphate; cGMP, cyclic guanosine monophosphate; PDE, phosphodiesterase; PKA, protein kinase A; PKG, protein kinase G

Under physiological conditions, cyclic adenosine monophosphate (cAMP) and cyclic guanosine monophosphate (cGMP) are metabolized by PDEs, a superfamily of enzymes (Soderling & Beavo 2000). Of note, eleven PDE families have been found to date, several of which are splice variants (Bolger 1994). PDE4, −7, and −8 are among the cAMP-specific enzymes. While PDE1, −2, −3, −10, and −11 employ both cyclic nucleotides, cGMP-specific PDEs are PDE5, −6, and −9 (Mehats et al. 2002). PDEs are involved in various processes, including the synthesis and action of pro-inflammatory mediators, ion channel function, muscle contraction, learning, differentiation, apoptosis, lipogenesis, glycogenolysis, and gluconeogenesis (Perry & Higgs 1998). PDEs have gained recognition as crucial pharmacological targets for the treatment of several illnesses, including heart failure, depression, asthma, inflammation, and erectile dysfunction, since they are vital regulators of cyclic nucleotide signaling with a variety of physiological activities (Rotella 2002). The effects of many extracellular signals, such as hormones, light, and neurotransmitters, are transduced via the ubiquitous second messengers cAMP and cGMP. Adenylyl cyclase and guanylyl cyclase catalyze the formation of these cyclic nucleotides from ATP and GTP, respectively. Forskolin can activate adenylyl cyclase, while nitric oxide (NO) can stimulate guanylyl cyclase. These enzymes can also be indirectly triggered by cell-surface receptors, such as the prostaglandin E2 receptor and the β-adrenoreceptor (Torphy 1998). The cyclic nucleotides bind to and activate their target enzymes, protein kinase A (PKA) and protein kinase G (PKG), when their intracellular concentrations increase (Krebs & Beavo 1979). These protein kinases phosphorylate substrates that control essential physiological processes, including transcription factors, ion channels, and contractile proteins. Phosphorylation modifies these substrates’ activity, which in turn modifies cellular activity. The active status of these pathways will obviously vary if the rate of cyclic nucleotide synthesis or breakdown is altered (Krebs & Beavo 1979) (Fig. 2).

**Fig. 3:**
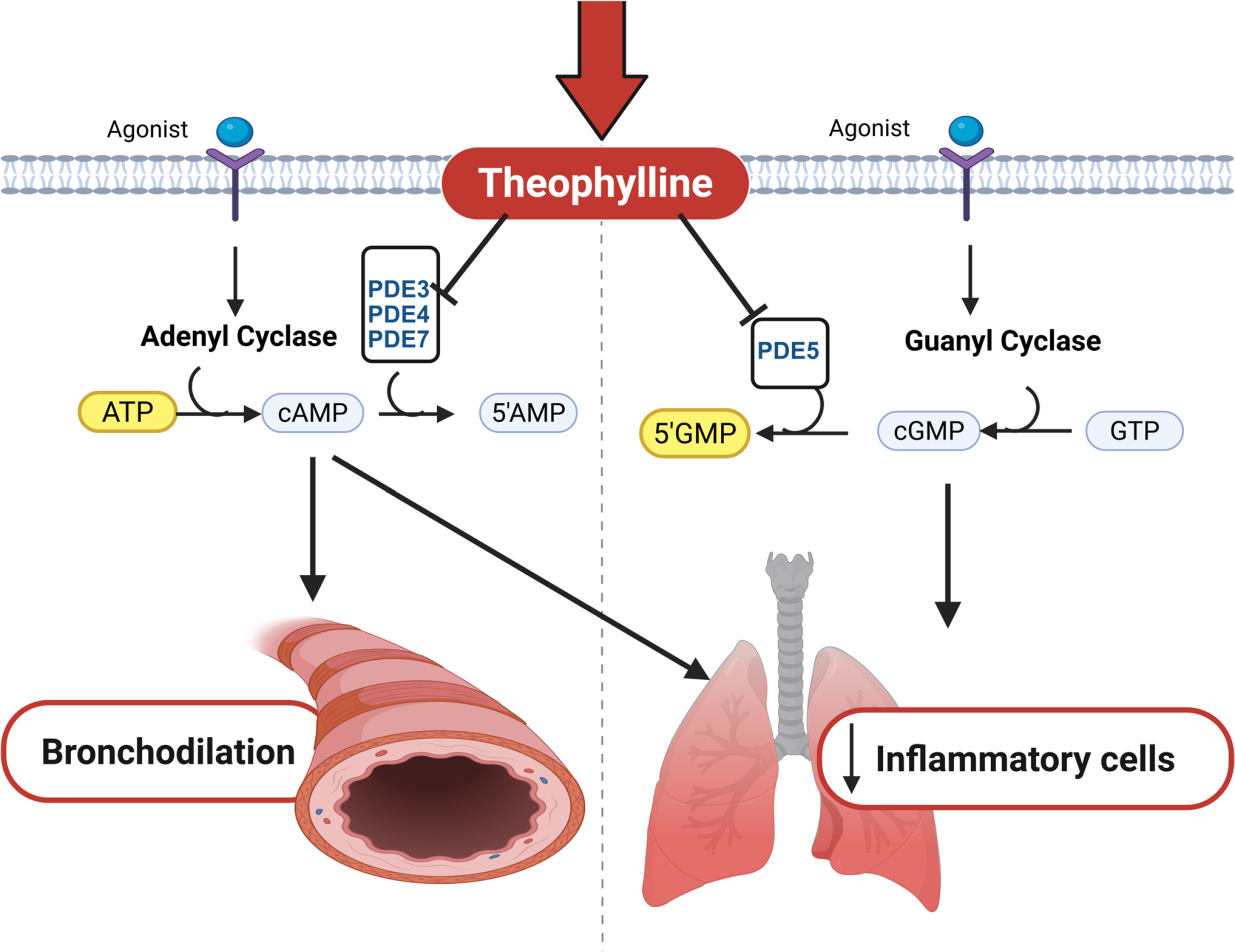
Mechanism of action of theophylline through modulation of cyclic nucleotide signaling. Theophylline inhibits phosphodiesterases (PDEs), thereby reducing the hydrolysis of cyclic AMP (cAMP) to 5′-AMP and cyclic GMP (cGMP) to 5′-GMP. Increased intracellular cAMP levels contribute to airway smooth muscle relaxation and bronchodilation, while modulation of cyclic nucleotide signaling in inflammatory cells reduces inflammatory responses. Abbreviations: cAMP, cyclic adenosine monophosphate; cGMP, cyclic guanosine monophosphate; PDE, phosphodiesterase

By the late 1970 s and early 1980 s, it was evident that a range of small chemical compounds could selectively block kinetically unique PDEs (Levin & Weiss 1976). Currently, four medications are on PDE3, and three are on PDE5. Two PDE4 inhibitor medication candidates are seeking approval. Hundreds of drugs are currently in the discovery phases, and approximately 20 PDE4 inhibitors are under clinical investigation (Xu et al. 2000). Indeed, theophylline has been used for many years as an asthma therapeutic agent based on its bronchodilatory and anti-inflammatory properties. It is an orally active non-selective PDE inhibitor. Still, other pertinent pharmacologic activities are likely to contribute to its efficacy, including the inhibition of phosphoinositide 3-kinase-δ, adenosine receptor antagonism, and enhancement of activity of certain histone deacetylases that deacetylate lysine residues in chromatin, thereby silencing gene transcription (Matera et al. 2017). In this context, the disadvantageous side-effect profile of theophylline has contributed, at least in part, to its decreased popularity as a medication and to a search for safer, more potent PDE inhibitors (V. Boswell-Smith et al. 2006a, b) (Fig. 3).

## The role of PDEs in asthma

A wide range of PDE isoenzymes can be pharmacologically targeted to generate distinct and beneficial biological effects, as many identified PDE isoforms exhibit unique and non-redundant physiological functions (MarshallRaj & Priyadarshini 2015). As such, the contribution of individual PDE variants must be understood within the broader context of an integrated and highly regulated signaling network, rather than through the lens of a single-enzyme model (Page & Cazzola 2014). Functionally, PDEs are expressed in a wide array of structural and immune cells, including fibroblasts, airway and vascular smooth muscle, epithelial cells, and numerous inflammatory cell types such as eosinophils, neutrophils, monocytes, macrophages, and T- and B-lymphocytes (Zuo et al. 2019). In asthma, cAMP represents a particularly important second messenger. It modulates the function of almost every cell type involved in the disease. It governs airway smooth muscle (ASM) tone via the β2-adrenergic receptor (β2-AR)–soluble adenylyl cyclase (sAC)–cAMP signaling axis (Jin et al. 2010). Elevating intracellular cAMP induces ASM relaxation and suppresses multiple immune and inflammatory processes, including T-cell activation and proliferation, eosinophil superoxide production, and chemotactic responses to inflammatory mediators (Page & Spina 2012). Increased cAMP also enhances mucociliary clearance (MCC) directly by accelerating ciliary beat frequency, which is regulated by the interplay between intracellular calcium and cAMP, and indirectly by attenuating inflammation triggered by allergens and other stimuli (Joskova et al. 2020). This modulation is clinically relevant, as ciliary dysfunction is a hallmark of moderate-to-severe asthma (Thomas et al. 2010). Moreover, cGMP is an essential second messenger in asthma because it regulates vascular smooth muscle relaxation via the NO-sGC-cGMP pathway (Stasch et al. 2011). As well, cGMP exerts a bronchodilator effect comparable to that of formoterol via activation of the AC/cAMP/PKA pathway (Lam & Bourke 2020). Recently, most human ASM cells derived from severe asthmatic donors have been found to possess a sGC that is either wholly or almost insensitive to NO and thus defective in NO-sGC-cGMP signaling (Ghosh et al. 2021). Transcriptomic data from selective expression of 18 of 24 PDE genes in human ASM cells from donors with and without asthma, as well as from donors with fatal asthma, indicate the presence of all PDE isoenzymes except PDE2 (Johnstone et al. 2018). PDE3, PDE4, PDE5, and PDE7 are also suggested to possibly play a part in asthma based on experimental studies. Moreover, there is documentation of PDE8 expression and function in ASM (Johnstone et al. 2018). The most common PDE isoenzyme in ASM is PDE3, the inhibition of which results in both ASM relaxation and increased relaxation induced by β2-AR stimulation (Mokry & Mokra 2013).

In allergic airway models, PDE3 deficiency has been shown to increase airway mucosal barrier function and decrease allergic airway inflammation (KleinJan 2016). There is proof that this isoenzyme is also vital for the degranulation of mast cells and basophils (Beute et al. 2020). The most essential isoenzyme present in the majority of inflammatory cells, PDE4, is critical to asthma pathophysiology. It may degrade cAMP and is highly expressed in inflammatory cells, including neutrophils, T cells, macrophages, and eosinophils (Mokry & Mokra 2013). Although its inhibition has not demonstrated acute bronchodilator effects in man, it is also present in ASM cells (Victoria Boswell-Smith et al. 2006a, b). The ciliary epithelia also exhibit high PDE4 activity (Joskova et al. 2020). Moreover, PDE3 and PDE4 are highly expressed in human lung microvascular endothelial cells and play a key role in leukocyte recruitment during pulmonary inflammation by regulating the expression of cell adhesion molecules (CAM) (Mokry & Mokra 2013).

Although PDE5 is widely expressed in ASM, bronchial epithelial cells, and lung fibroblasts, it is most highly expressed in vascular smooth muscle cells (Zuo et al. 2019). Protein kinase G-dependent smooth muscle relaxation was induced by PDE5 inhibition and the resultant increase in cGMP, with no detectable effect on cAMP (Nijkamp & Folkerts 1995). And a bronchodilatory effect *in vivo* and an *in vitro* relaxation of the airway in guinea pigs (Kapui et al. 1999). Immune responses were similarly inhibited by increased intracellular cGMP (Boswell‐Smith et al. 2006). Decreased circulating leukocyte and eosinophil counts and lung homogenate levels of interleukin (IL)−4, IL-5, and TNF-α were associated with the profound suppression of both *in vivo* and *in vitro* contractile responses to cumulative doses of acetylcholine and histamine in ovalbumen-sensitized animals (Urbanova et al. 2017). However, Banner and Page were unable to demonstrate any significant effects of either acute or chronic PDE5 inhibitor treatment on ovalbumen-induced eosinophil infiltration in guinea pigs (Banner & Page 1995). PDE7 modulates intracellular cAMP levels and is present in the soluble fraction of CD4 + and CD8 + T lymphocytes (Giembycz et al. 1996). Some theories suggest that PDE7 may play an essential role in T cell activity (Nakata et al. 2002).

Nevertheless, in a model of asthma using sensitized mice, it was determined that PDE7 does not relate to airway inflammation or hyperreactivity (Chevalier et al. 2012). Although PDE8 is less frequently expressed compared to PDE4, it has a 40–100-fold higher affinity for cAMP (Vang et al. 2016). It is targeted in lipid rafts and selectively regulates β2-AR-stimulated cAMP signalling but does not affect prostaglandin E2-stimulated cAMP signalling in ASM (Zuo et al. 2019). PDE8 has been suggested as a potential target to inhibit the migration of activated T-lymphocytes from the circulation into tissues during the inflammatory response (Vang et al. 2010). These findings highlighted that the PDE family could be a drug target for the treatment of asthma, as well as the potential to develop drugs that could both inhibit individual PDEs and interact with multiple PDEs simultaneously (Page & Cazzola 2014).

The fact that some PDEs are sensitive to short-term allosteric modulation by endogenous activators or inhibitors further complicates the functional roles of different PDEs in intact tissues (Manganiello et al. 1991). For example, Ca21/calmodulin allosterically activates PDE1. Thus, drugs that promote Ca21 mobilization and, as a consequence, increase cytosolic Ca21/calmodulin concentration activate PDE1 in intact cells (Tanner et al. 1986). In intact cells, an increase in cyclic GMP content allosterically activates PDE2, leading to a reciprocal decrease in cyclic AMP levels (Whalin et al. 1991). However, hydrolysis of cyclic AMP by PDE3 is competitively inhibited by cyclic GMP (Harrison et al. 1986). This appears to be the mechanism by which agents that increase cyclic GMP levels in platelets and vascular smooth muscle also increase cyclic AMP levels (Maurice et al. 1991). In the presence of isozyme-selective PDE inhibitors, this mechanism may also help determine the sensitivity of human airway smooth muscle to inhibitory non-adrenergic-non-cholinergic (iNANC) stimulation (Fernandes et al. 1994). Another short-term PDE-regulating mechanism is protein phosphorylation. Protein kinase A phosphorylates PDE1A1 and PDE1A2, while calmodulin kinase II phosphorylates PDE1B (Hashimoto et al. 1989). Phosphorylation always lowers the enzyme’s affinity for Ca2 +, thereby reducing the enzyme’s Ca2 + sensitivity. PKA uses both PDE3 subtypes as substrates, while insulin-sensitive protein kinases also use PDE3B as a substrate (Manganiello et al. 1991). This enzyme’s activity is increased when either kinase phosphorylates it. The PKA route activates only specific subtypes of PDE4D that harbor a PKA phosphorylation consensus site in the N-terminal domain. Differential regulation within a single PDE subtype is therefore made possible. Lastly, PKA and protein kinase G both phosphorylate PDE5 (Thomas et al. 1990). Although the functional effects of phosphorylation are unknown, it could boost enzyme activity (Burns et al. 1992).

## The potential role of PDE inhibitors in asthma

It has been illustrated that PDE inhibitors produce beneficial effects in the management of asthma through different pathways that affect numerous components of asthma, including airway smooth muscle cells, the MCC, lung vascular permeability, inflammatory cells, and others.

### Effects of PDE inhibitors on airway smooth muscle cells

Increased PDE activity, specifically PDE4 and PDE3, is found in asthmatic airway smooth muscle and immune cells (Keir & Page 2014). This inhibits signaling required for bronchodilation and inflammatory regulation and accelerates the breakdown of cAMP. Inhibition of these enzymes restores cAMP signaling and affects all airway tissues (Keir & Page 2014). Inhibiting PDE4 or PDE3 leads to PKA activation and increases intracellular cAMP levels. This activation decreases myosin light-chain phosphorylation and calcium release in airway smooth muscle, enhancing bronchodilation and decreasing contractility (Keir & Page 2014). Asthmatic airway smooth-muscle cells become less reactive to β2-agonists because of fast cAMP breakdown, which upregulates PDE4D; inhibition of PDE4 restores this response (Keir & Page 2014).

Human airway smooth-muscle cells express different PDE isoforms, including PDE3, PDE4B, and PDE4D (Billington et al. 2008). PDE4 inhibitors have been demonstrated in some studies to relax the intrinsic tone of isolated human bronchial muscle (Naline et al. 1996). This splice variant was identified as the major physiological regulator of β2-adrenoceptor-induced cAMP turnover in human airway smooth muscle using siRNA targeting PDE4D5 (Billington et al. 2008). The PDE4 inhibitor CHF-6001, and to a lesser extent, roflumilast, inhibit bronchoconstriction-induced remodeling in guinea pig precision-cut lung slices [51]. Ensifentrine (RPL554), an inhaled bifunctional dual phosphodiesterase 3/4 inhibitor that exhibits both bronchodilator and anti-inflammatory activities, provides a new option for the treatment of COPD and other inflammatory airway diseases, including asthma [51]. A study using PDE4D –/– mice demonstrated a marked impairment of airway smooth-muscle contractility in isolated trachea, characterized by a profound decrease in maximal tension and reduced responsiveness to muscarinic cholinergic agonists (Méhats et al. 2003). This provides further evidence that PDE4D is a key component of the contractile response of airway smooth muscle.

Additionally, olprinone, a PDE3 inhibitor, had a dose-dependent antagonistic effect on methacholine-induced bronchoconstriction in dogs, indicating a significant role for PDE3 in airway smooth muscle contraction (Hirota et al. 2001). In addition, the naturally derived PDE4-selective inhibitor quercetin relaxes airway smooth muscle via cAMP-mediated pathways and augments β-agonist relaxation in tracheal rings from male A/J mice. Quercetin directly attenuated phospholipase C activity, decreased inositol phosphate synthesis, and decreased intracellular calcium responses to Gq-coupled agonists (Townsend & Emala 2013). These findings highlighted that the natural PDE4-selective inhibitor quercetin may afford therapeutic relief of asthma symptoms and decrease reliance on short-acting β-agonists. Importantly, airway remodeling is a pathological feature of chronic asthma and involves both lung structural and immune cells. One of the main contributors to airway remodeling is airway smooth muscle, which is thickened in asthma and produces more extracellular matrix (ECM).

Notably, cAMP has been shown to contribute to airway smooth muscle relaxation and to anti-remodeling effects in airway smooth muscle cells (Wójcik-Pszczoła et al. 2019). Selective inhibition of PDE1, PDE3, PDE4, and PDE7 reduces proliferation, contractility, ECM protein expression, and migration compared with non-selective and selective PDE inhibitors (Wójcik-Pszczoła et al. 2019). Consistently, findings from a pre-clinical study showed that β2-adrenoceptor agonist formoterol and PDE inhibitors IBMX, aminophylline, and roflumilast induced cAMP efflux and concentration-dependent relaxation of rat trachea pre-contracted with carbachol. Pretreatment of trachea with MK-571 (MRP transporter inhibitor), AMP-CP (ecto-5′NT inhibitor), or CGS-15943 (non-selective adenosine receptor antagonist) potentiated the relaxation induced by β_2_-adrenoceptor agonists. Still, it did not change the relaxation induced by PDE inhibitors (Satori et al. 2023).

While some studies have demonstrated that PDE4 inhibitors relax inherent tone in isolated human bronchial muscle (Schmidt et al. 2000), others have shown that PDE3 or PDE4 inhibitors alone do not, but that, in combination, they relax inherent tone (Rabe et al. 1993). Moreover, PDE3 or PDE4 inhibition alone did not alter contraction of human airway smooth muscle to allergens or LTC4, but together they acted in concert to prevent contraction (Schmidt et al. 2000). Notably, human bronchial smooth muscle pre-contracted by a variety of contractile agents may be effectively relaxed by the dual PDE3/4 inhibitor RPL 554 (Calzetta et al. 2013). It has been demonstrated to act similarly in concert with anti-cholinergic and β2 agonists in guinea pig lung *in vivo* (Calzetta et al. 2013) and to act in concert with muscarinic receptor antagonists and, to a lesser extent, with β2 agonists to relax human bronchial smooth muscle (Calzetta et al. 2013) and small airways (Calzetta et al. 2013). Furthermore, the non-selective PDE inhibitor theophylline, which is commonly used in the management of asthma, has anti-inflammatory activity (Ghosh et al. 2024). Furthermore, the dual PDE3/PDE4 inhibitors tanimilast and ensifentrine have been shown to improve lung function, reduce exacerbations, and enhance quality of life in COPD patients by inducing bronchodilation (M. Cazzola et al. 2024a, b). However, it has not yet been determined which type of COPD patient might benefit more from inhaled PDE4 inhibitors, and it remains unclear whether concomitant inhibition of PDE3 and PDE4 confers a significant benefit compared with PDE4 inhibition alone.

**Fig. 4:**
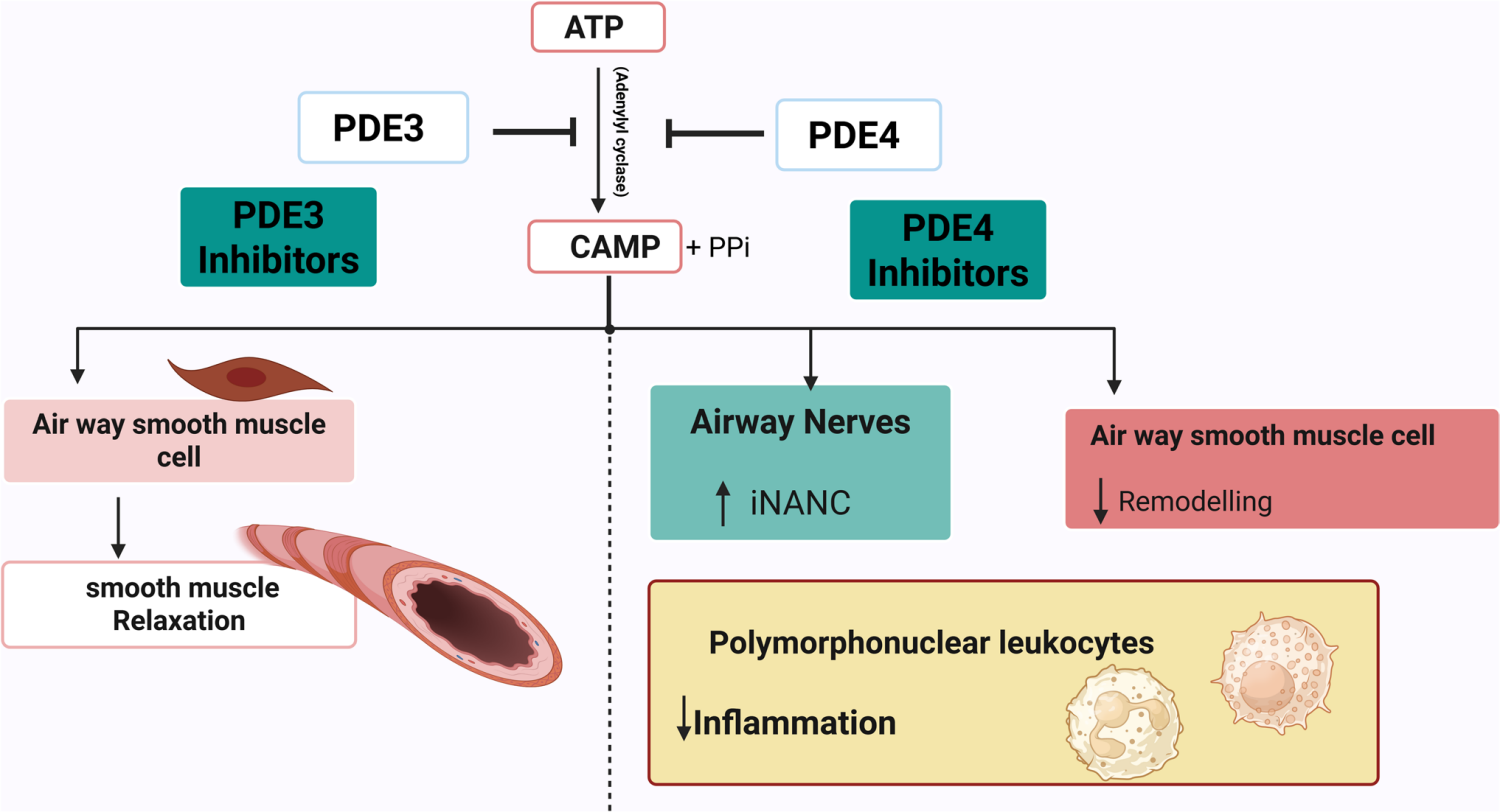
Schematic representation of the role of PDE3 and PDE4 inhibition in regulating intracellular cAMP signaling in the airways. Cyclic AMP (cAMP) is generated from ATP by adenylyl cyclase (ATP → cAMP + PPi), and its accumulation is enhanced by inhibition of PDE3 and/or PDE4. Increased cAMP contributes to airway smooth muscle relaxation and bronchodilation, enhances inhibitory non-adrenergic, non-cholinergic (iNANC) neural activity, reduces airway smooth muscle remodeling, and suppresses inflammatory responses in polymorphonuclear leukocytes. Abbreviations: cAMP, cyclic adenosine monophosphate; iNANC, inhibitory non-adrenergic, non-cholinergic; PDE, phosphodiesterase; PPi, pyrophosphate

Clinically, a randomized clinical trial showed that a novel inhaled dual PDE3 and PDE4 inhibitor, RPL554, acts as a bronchodilator and anti-inflammatory drug in patients with asthma (Franciosi et al. 2013). In addition, nebulized ensifentrine (RPL554) results in a dose-dependent bronchodilatory effect and does not cause the characteristic β_2_-agonist systemic adverse effects (Bjermer et al. 2019). Therefore, ensifentrine may be safer than β_2_-agonists in asthmatic patients with cardiovascular diseases. Another clinical trial showed that the inhaled PDE4 inhibitor CHF6001 attenuates the late asthmatic response, with a greater reduction in sputum eosinophil counts. However, these changes were not statistically significant compared with placebo (Singh et al. 2016). Therefore, this inhaled PDE4 inhibitor may potentially provide clinical benefits in patients with atopic asthma. Conversely, a randomized, double-masked, placebo-controlled trial of 24 weeks of roflumilast versus placebo showed no benefit in asthmatic patients. It may exacerbate bronchoconstriction in obese persons with uncontrolled asthma (Dixon et al. 2023). These outcomes suggest the significance of studying interventions in diverse subpopulations of people with asthma, chiefly people with obesity and asthma who may respond differently to medications than lean people with asthma. Weight loss of at least 5% was associated with improved asthma control, indicating that interventions other than roflumilast that promote weight loss may be effective for the treatment of poorly controlled asthma in people with obesity. The mechanism for the increased risk of exacerbations by roflumilast was not elucidated. However, the non-selective PDE inhibitor theophylline causes asthma exacerbations in obese asthmatic patients than in lean people, which might be due to paradoxical effects in obese people with asthma (Dixon et al. 2006). These findings highlighted that PDE inhibitors may be effective in the management of asthma by modulating airway smooth muscle cell responses (Figs. 3 and 4).

### Effects of PDE inhibitors on the MCC

The MCC represents one of the most important defense airway mechanisms. Notably, excessive mucus production and persistent cough are well-recognized features of severe asthma and are also associated with defects in MCC. Damaged airway epithelium and impaired ciliary cell function lead to MCC dysfunction, increasing susceptibility to infection and inflammation (Jesenak et al. 2023). It has been demonstrated that MCC dysfunctions in asthma are characterized by intracellular disorientation, abnormal cilia, and cytoplasmic blebs (Jesenak et al. 2023). Therefore, restoration of MCC dysfunction could represent a new therapeutic approach for the management of asthma and other chronic respiratory diseases. Prominently, cystic fibrosis transmembrane conductance regulator (CFTR) is a crucial gene and protein that acts as a channel, transporting chloride ions and water across cell membranes, vital for thin, flowing mucus in organs like the lungs, pancreas, and sweat glands; mutations in the CFTR gene cause cystic fibrosis (CF) (90). It has been shown that acquired CFTR failure can lead to impaired MCC, independent of the congenital CFTR dysfunction seen in patients with cystic fibrosis (Liu et al. 2005). CFTR is the predominant cAMP-activated chloride channel on the apical membrane of airway epithelia. *In vitro* and *in vivo*, smoke-induced CFTR failure has been associated with the pathophysiology of COPD by predisposing to mucociliary retention, epithelial dysfunction, and chronic bronchitis. In an epithelial cell line, PDE4 and PDE3 inhibition were reported to enhance CFTR-mediated chloride secretion (Liu et al. 2005), suggesting that PDE3/4 inhibitors could further benefit patients with COPD by enhancing MCC. Originally, it was established that roflumilast restored CFTR function in cigarette smoke-exposed human bronchial epithelial cells (Lambert et al. 2014), and this effect was further augmented by ivacaftor, the newly licensed CFTR corrector drug.

**Fig. 5:**
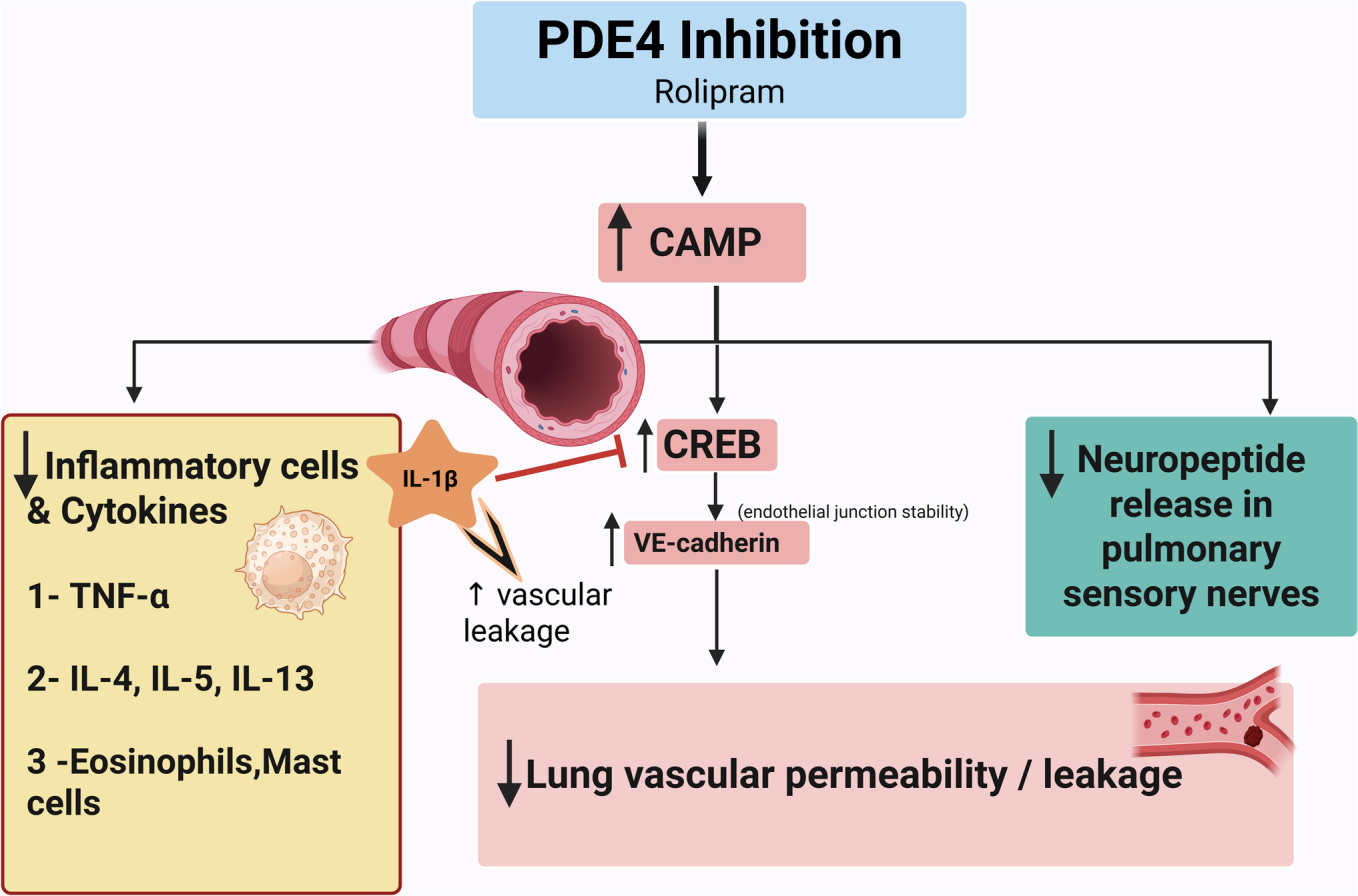
Mechanisms by which phosphodiesterase-4 (PDE4) inhibition modulates airway inflammation, neurogenic responses, and lung vascular permeability. PDE4 inhibition (e.g., rolipram) increases intracellular cyclic AMP (cAMP) levels, resulting in suppression of inflammatory cells and cytokine production (including TNF-α, IL-4, IL-5, and IL-13) and reduction of eosinophil- and mast cell–driven airway inflammation. Elevated cAMP also decreases neuropeptide release from pulmonary sensory nerves. Importantly, PDE4 inhibition contributes to preservation of endothelial barrier function by enhancing cAMP-dependent activation of cAMP response element-binding protein (CREB), which promotes vascular endothelial (VE)-cadherin expression and stabilizes endothelial junction integrity, thereby reducing lung vascular permeability and vascular leakage. In contrast, inflammatory signaling such as IL-1β can suppress CREB/VE-cadherin signaling and increase vascular leakage. This mechanistic pathway is supported by experimental findings demonstrating that rolipram prevents endotoxemia-induced lung vascular leakage via CREB-mediated regulation of VE-cadherin (Xiong et al. 2020). Abbreviations: cAMP, cyclic adenosine monophosphate; CREB, cAMP response element-binding protein; IL, interleukin; PDE4, phosphodiesterase-4; TNF-α, tumor necrosis factor-alpha; VE-cadherin, vascular endothelial cadherin

Furthermore, roflumilast enhances the depleted airway surface liquid depth in smoke-exposed human airway epithelial monolayers. In addition, CFTR-dependent fluid secretion induced by roflumilast in the murine intestine enhanced the diameter of ligated murine intestinal segments as well as the wet/dry ratio (Lambert et al. 2014). This effect has been postulated to underlie the clinically observed diarrhea in some patients treated with roflumilast. Moreover, PDE3 and PDE4 inhibitors accelerate ciliary beat frequency in upper and lower airway tissue *in vitro* (Cervin & Lindgren 1998). In addition, roflumilast suppresses a respiratory syncytial virus-induced increase in MUC5AC (Mata et al. 2013). PDE inhibition has a similar impact on epithelial function, and increasing cAMP signaling enhances mucus clearance and ciliary beat frequency (Van Ly et al. 2013). This reduces mucus retention caused by allergic irritation. Although systemic side effects of oral PDE4 inhibitors continue to limit clinical use, these pathways provide a rationale for targeting the PDE isoenzymes as a therapeutic strategy in asthma (Beghè et al. 2013). Increased inflammation is associated with lower MCC in patients with asthma compared with healthy controls. High rates of MCC in mild asthma may indicate a compensatory mechanism present in mild disease but lost with high levels of inflammation (Corcoran et al. 2021). Previous studies of MCC in asthma have yielded inconsistent results, likely because of the heterogeneous nature of the disease. The presence or absence of type 2 inflammation provides one means to categorize patients with asthma and has previously been shown to predict therapeutic response and outcomes (Ray et al. 2020). Therefore, restoration of the MCC by PDE inhibitors may reduce the severity and improve the clinical outcomes in patients with asthma (Fig. 5).

### Effects of PDE inhibitors on lung vascular permeability

Of note, injury to lung fibroblasts, epithelial cells, and vascular endothelial cells contributes to the development of lung vascular permeability in asthma (Harkness et al. 2014). A case–control study revealed that bronchial vascular leakage is the primary pathophysiological feature underlying the deterioration of asthma before cellular inflammation (Khor et al. 2009). Besides, PDE4B is implicated in the development of acute lung injury (ALI) by inducing airway vascular permeability via NF-κB activation in the LPS-induced ALI model (Ma et al. 2014). Consistently, deletion of the *PDE4B* gene attenuates LPS-induced ALI, suggesting that inhibition of PDE4B may reduce ALI and vascular leakage in respiratory diseases (Ma et al. 2014). Importantly, PDE4 inhibition may also influence pulmonary endothelial barrier function and lung vascular permeability. Inflammatory signaling, such as IL-1β, has been shown to suppress the cAMP response element-binding protein (CREB) pathway, resulting in reduced vascular endothelial (VE)-cadherin expression and destabilization of endothelial adherens junctions, thereby promoting lung vascular leakage and inflammatory lung injury (Xiong et al. 2020).

In contrast, PDE4 inhibition by rolipram increases intracellular cAMP levels, restores CREB activation, and enhances VE-cadherin expression, ultimately preserving endothelial junction integrity and reducing endotoxemia-induced pulmonary vascular leakage (Xiong et al. 2020). Kosutova et al. found that roflumilast attenuates ALI by inhibiting lung inflammation and airway vascular permeability in animal models. Treatment with roflumilast abridged the leak of cells, predominantly of neutrophils, into the lung, decreased concentrations of cytokines and oxidative products in the lung and plasma, and reduced lung cell apoptosis and edema formation (Kosutova et al. 2018). Henceforth, suppression of lung vascular permeability by PDE inhibitors may be useful in the management of asthma.

### Effects of PDE inhibitors on inflammatory cells

Indeed, inflammatory cells, mainly neutrophils and eosinophils, are involved in the pathogenesis of asthma. Notably, predominant eosinophilic, neutrophilic, or mixed eosinophilic/neutrophilic inflammatory patterns are associated with asthma (Duchesne et al. 2022). In particular, eosinophilic inflammation is associated with the full range of asthma severity, whereas neutrophilic inflammation typically occurs in more severe asthma (Ji & Li 2023). Eosinophilic asthma includes both allergic and non-allergic phenotypes, with immune responses mediated by T helper (Th)2 cell-derived cytokines, whilst neutrophilic asthma is mostly dependent on Th17 cell-induced mechanisms. These immune-inflammatory profiles develop as a consequence of a functional impairment of T regulatory (Treg) lymphocytes, which promotes the activation of dendritic cells directing the differentiation of distinct Th cell subsets (Duchesne et al. 2022; Ji & Li 2023). It has been observed that the expression of PDE4 and PDE7 is augmented in immune cells isolated from asthmatic patients, suggesting immunological effects of PDEs in asthma pathogenesis (Jones et al. 2007). Furthermore, roflumilast, by inhibiting PDE7 expression in immune and inflammatory cells, can reduce inflammation in asthma and COPD (Cazzola et al. 2016). Roflumilast can directly and indirectly act on inflammatory cells, such as eosinophils and neutrophils, and can also improve airway inflammation by promoting salbutamol-induced clearance of inflammatory mediators in the airway or through other approaches, and by affecting the levels of certain inflammatory mediators (Mendes et al. 2017).

In immune cells, PDE4 inhibition diminishes T-cell activation, mast cell mediator release, eosinophil recruitment, and macrophage cytokine production, all of which reduce airway hyperresponsiveness (Sun et al. 2006). Furthermore, selective and dual PDE3/4 inhibitors improve lung function in experimental asthma, along with reduced inflammatory-cell infiltration and goblet-cell hyperplasia (Sherpa et al. 2025; Sun et al. 2006; Y. Zhang et al. 2024a, b, c). Hence, PDE inhibitors, by augmenting cAMP-PKA signaling, inhibit pro-inflammatory pathways and improve airway function both *in vitro* and *in vivo* (Y. Zhang et al. 2024a, b, c). Specifically, a compound isolated from several traditional Chinese medicines, nobiletin, exerts an anti-asthmatic effect. It alleviates airway hyperresponsiveness in mice by activating the cAMP-PKA-CREB signaling pathway downstream of PDE4B in mouse lung tissues and RAW264.7 cells (Y. Zhang et al. 2024a, b, c), indicating that nobiletin exerts anti-asthmatic activity by targeting PDE4B. In addition, activation of PKA increases the phosphorylation of CREB, which inhibits NF-κB activity and reduces inflammation (Christian et al. 2016). The CREB pathway is also activated by elevating cAMP levels. In cells activated by TNF-α, this increases MKP-1 and decreases the levels of inflammatory mediators, such as IL-8 (Patel et al. 2015). As NF-κB is critical for Th2 cell differentiation, inhibition of the NF-κB p65 nuclear entry response could also alleviate asthma symptoms via nobiletin, acting through the cAMP-PKA-CREB and NF-κB signaling pathways by inhibiting PDE4B (Y. Zhang et al. 2024a, b, c). Moreover, TNF and TNF ligand superfamily member 11 (TNFSF11) and its known receptor, TNF receptor superfamily 11 A (TNFRSF11A), have been implicated in asthma; however, the underlying mechanisms remain unknown (D. Zhang, J. Zhang, Q. Qi, et al., 2024). The expression of the remodeling proteins induced by TNFSF11 meaningfully reduced after pretreatment with the STAT3 inhibitor in HBE cells (D. Zhang, J. Zhang, Q. Qi, et al., 2024). The above results also showed that blocking TNFSF11 with denosumab alleviated airway remodeling via the TGFβ1/STAT3 signaling pathway in humanized HSC-NOG-EXL mice with HDM-induced asthma. In addition, TNF-like cytokine 1 A (TL1A), which exists as membrane-bound (mTL1A) and soluble (sTL1A) forms, is upregulated in asthma (Zhang et al. 2022). Interestingly, syndecan-1 is a transmembrane proteoglycan of heparin sulfate that can regulate various cell signaling pathways in airway epithelial cells and fibroblasts. Findings from a pre-clinical study showed that overexpression of syndecan-1 is correlated with TGFβ1/Smad3-mediated airway remodeling *in vivo* and *in vitro* (Zhang et al. 2021). However, evidence for the effects of PDE inhibitors on syndecan-1 expression and TL1A in asthma is limited. Importantly, neutrophilic asthma is steroid-resistant and characterized by the absence or suppression of the T-helper type II (TH2) pathway and by increased TH1 and/or TH17 pathways. The PDE4 inhibitor roflumilast has anti-inflammatory effects and may have a therapeutic role in neutrophilic asthma by reducing airway hyperresponsiveness and lung inflammation in a mouse model of neutrophilic asthma. Also, additive effects were observed when dexamethasone was added to roflumilast treatment, possibly due to the recovery of HDAC2/β-actin activity (Fernandes et al. 1994). Therefore, PDE inhibitors, by their anti-inflammatory effect, can attenuate TNF-induced inflammation in asthma.

### Other effects of PDE inhibitors in asthma

Furthermore, treatment with PDE inhibitors may improve clinical outcomes in asthma–chronic pulmonary overlap (ACO) through other mechanisms. PDE inhibitors improve lung ventilation function and FEV1 (Padrão et al. 2018). For example, roflumilast increases FEV1 to 53.52 mL in patients with COPD (Bateman et al. 2015). Interestingly, roflumilast improves pulmonary ventilation in ACO in a dose-dependent manner (Bateman et al. 2010). Moreover, PDE inhibitors mitigate bronchial remodeling and stiffness, which are common in ACO. Bronchial remodeling in asthma is due to long-term inflammation and the deposition of fibronectin (Louw et al. 2007). In this state, PDE inhibitors such as roflumilast reduce bronchial inflammation and fibrosis by increasing intracellular cAMP, thereby attenuating the proliferation of bronchial myofibroblasts (Yalcin et al. 2016). A recent systematic review and meta-analysis highlighted that roflumilast showed a modest improvement and had an insignificant effect on FEV1, which might be due to heterogeneity across the selected studies or differences in dose and duration. Moreover, nerandomilast, a PDE4 inhibitor approved for the treatment of idiopathic pulmonary fibrosis and progressive pulmonary fibrosis, may be effective in the management of asthma (Fernandes et al. 1994).

Additionally, rolipram, a selective PDE4 inhibitor, has been investigated for its effectiveness in asthma. Pretreatment with rolipram significantly reduced lung resistance, eosinophil infiltration, and histamine release into the bronchoalveolar space during the early asthmatic reaction. These effects were generally comparable with those of dexamethasone, except that dexamethasone also reduced the influx of neutrophils into bronchoalveolar lavage fluid (Fernandes et al. 1994).

Importantly, mitochondrial dysfunction and oxidative stress are involved in the pathogenesis of asthma and related metabolic disorders (Hartsoe et al. 2024). Therefore, mitochondrial DNA levels are elevated in asthmatic patients compared with healthy controls (Carpagnano et al. 2016). Thus, restoring mitochondrial function can prevent oxidative stress-induced asthma. Many studies have confirmed that PDE inhibitors, by regulating cAMP/cGMP, regulate mitochondrial bioenergetics and suppress oxidative stress (Corum et al. 2020; Tetsi et al. 2017).

## Efficacy of PDE inhibitors as asthma treatment in clinical settings

Of note, the non-selective PDE inhibitors, such as theophylline and its derivatives (aminophylline, oxtriphylline, and dyphylline), are commonly approved for the management of asthma and COPD. Large doses of theophylline are effective in the management of asthma and COPD but are associated with serious adverse effects (Mario Cazzola et al. 2024a, b; Ghosh et al. 2024). Therefore, a low dose of theophylline may be effective in treating bronchospasm in asthma and COPD without serious adverse effects. However, a randomized, placebo-controlled clinical trial found that a low dose of theophylline was ineffective in treating acute exacerbation and bronchospasm in patients with either asthma or COPD compared with placebo (Devereux et al. 2018). Regarding the clinical efficacy of roflumilast, a clinical study showed that roflumilast improves FEV1 and clinical outcomes in asthmatic patients compared with placebo, suggesting that roflumilast could be an effective adjuvant treatment for asthma (Meltzer et al. 2015). Consistently, a pooled analysis of multiple clinical studies found that roflumilast was well tolerated, with few adverse effects, in asthmatic patients (Chervinsky et al. 2015). However, a recent systematic review and meta-analysis highlighted that weight loss and gastrointestinal adverse effects are frequent in asthmatic patients with obesity, resulting in an increased rate of discontinuation of roflumilast use. Nevertheless, subgroup analysis illustrated that asthmatic patients with corticosteroid resistance and persistent airway inflammation limit the clinical efficacy of roflumilast (Alruwaili et al. 2025). Thus, heterogeneity among the selected studies and the limited number of clinical trials may limit the conclusion regarding the safety and clinical efficacy of roflumilast in asthma.

Furthermore, the potent PDE4 inhibitor CHF6001 has been reported to be well tolerated in humans and to inhibit allergen-induced immune responses in asthmatic patients compared with placebo (Singh et al. 2016). CHF6001 has potent anti-inflammatory effects with minimal adverse effects, comparable to those of other PDE inhibitors, in patients with chronic bronchitis (Singh et al. 2019). Similarly, the PDE4 inhibitor GSK256066 attenuates early and late immune responses with limited systemic adverse effects in asthmatic patients (Singh et al. 2010). A Phase IIa clinical trial tested the use of GSK256066 at different doses and found no significant differences in the management of asthma, suggesting that GSK256066 had a dose-independent anti-asthmatic effect (Singh et al. 2010).

Interestingly, the dual PDE3 and PDE4 inhibitor ensifentrina (RPL554) has good safety and efficacy, without cardiac adverse effects, in patients with asthma. Ensifentrina induces bronchodilation and is considered an effective adjuvant in the treatment of asthma and COPD (Singh et al. 2018, 2020). Despite the clinical efficacy of PDE inhibitors in the management of asthma and COPD, some clinical trials regarding PDE inhibitors such as cilomilast, MK0873, and revamilast were discontinued (Balasubramanian et al. 2016; Boot et al. 2008; Martina et al. 2006; Singh et al. 2020). These verdicts highlighted that PDE inhibitors have potential therapeutic efficacy with a good safety profile in asthma.

Overall, PDE inhibitors could be a therapeutic strategy in the management of asthma by modulating multiple signaling pathways.

## Discussion

PDE4 and dual PDE3/4 inhibitors, owing to their anti-inflammatory effects, have considerable interest as potential therapeutic agents for the treatment of respiratory diseases, including asthma. However, many of these agents have failed in early development for various reasons, including dose-limiting side effects when administered orally. The majority of failed agents are orally administered and non-selective (Corum et al. 2020). Nevertheless, inhaled dual PDE3/4 inhibitors may represent one strategy to improve the therapeutic index and exhibit enhanced efficacy compared with inhibition of either PDE3 or PDE4 alone (M. Cazzola et al. 2024a, b). PDE inhibitors could be useful in the treatment of asthma due to their bronchodilatory and/or anti-inflammatory effects. Although the potential therapeutic utility of PDE inhibitors has been demonstrated in various animal models of asthma, their clinical efficacy has been limited by dose-limiting side effects; no PDE inhibitor has yet been approved for the treatment of patients with asthma. Even though new PDE inhibitors have been synthesized, most data are from cellular and tissue-level studies, with human trials still on the horizon. Actually, only CHF6001, an inhaled PDE4 inhibitor, and RPL554, a dual PDE3/4 inhibitor, are still under clinical development (Matera et al. 2014). The complex and multifaceted roles of PDE inhibitors in relation to asthma are increasingly evident. The development of the rationale for targeting PDEs closely parallels the evolution from asthma characterization to the appreciation of its multiple molecular underpinnings. In the clinical setting, the evidence is scarce and largely limited to PDE4 inhibitors in COPD, showing a benefit in improving lung function and reducing the likelihood of exacerbations, but without a significant impact on quality of life, based on the results of a systematic review of clinical trials involving cilomilast, roflumilast, and tetomilast (Janjua et al. 2020). The main difficulties in their wider clinical use and development include a safety profile that negatively affects compliance among patients with severe asthma (Rabe & Watz 2017). In addition, several pharmacokinetic and formulation-related limitations remain critical barriers, including inadequate chemical stability, suboptimal membrane permeability, and limited bioavailability, which can restrict drug exposure at target airway compartments and contribute to variability in clinical response (Rabe & Watz 2017). A currently recruiting Phase 1 trial of roflumilast in patients with severe asthma could provide further insights into the clinical applicability of PDE4 inhibition in asthma. Moreover, two novel PDE inhibitors, ensifentrine and tanimilast, are currently in clinical trials for COPD and asthma (Bjermer et al. 2019).

Importantly, asthma is characterized by complex, cell-specific pathophysiologic mechanisms, and inhibiting PDEs elicits diverse cellular responses depending on the cell type involved, including airway smooth muscle relaxation, bronchodilation, and suppression of inflammatory pathways. In particular, inhibition of PDE4 and PDE5 has also been described to affect airway wall remodeling (Bjermer et al. 2019; Rabe & Watz 2017). In addition, the NOG-EXL mice model positively replicated key features of asthma, including inflammatory cell infiltration, collagen deposition, and increased mucous secretion. This humanized mouse model has the potential to be a valuable tool for studying asthma and for developing clinical treatment strategies (D. Zhang, J. Zhang, C. Xu, et al., 2024). Hence, the application of PDE inhibitors in this model may open a new avenue for elucidating the molecular mechanisms of these agents in asthma. The complexity of the control exerted by the PDE superfamily on cyclic nucleotide communication across a broad array of immunological and structural cells in the lung provides an attractive, if challenging, therapeutic target.

The rationale for PDE inhibition in asthma is based on increases in intracellular cAMP and, to a lesser extent, cGMP. These second messengers have master regulatory effects on ASM relaxation via cAMP, with simultaneous inhibition of immune cells implicated in the pathophysiology of asthma. PDE4, PDE3, PDE7, and PDE8 have non-redundant functions in compartmentalizing and regulating complex cyclic nucleotide gradients created by the expression of specific PDE isoenzymes in discrete cell types. Such compartmentalization is not without importance, with the predominance of PDE3 in ASM and reign of PDE4 in inflammatory cells suggesting that selective inhibition may have cell-type-specific effects. On the other hand, a single-target strategy is discouraged by evidence of overlapping and synergistic functions, as reflected by the greater efficacy of combination PDE3/4 inhibition in relaxing human bronchial smooth muscle compared with single-isoenzyme inhibition.

Moreover, clinical experience with PDE4 inhibitors, particularly in asthma, underlines both the benefits and drawbacks of this approach. However, the major disappointment has been the failure of several PDE4 inhibitors to gain approval for asthma, due mainly to dose-limiting gastrointestinal adverse effects, including nausea, vomiting, and diarrhea. Such side effects arise directly from the expression and crucial role of PDE4 in gastric and neurological tissues and reflect a fundamental challenge in drug development: achieving an adequate therapeutic index by differentiating systemic toxicity from desired pulmonary effects (McDonough et al. 2020). In light of this experience, there is reason to consider that sophisticated approaches to asthma may be more effective than diffuse PDE4 inhibition throughout the body. There are several different ways to evaluate these strategies. First, it makes sense to develop inhaled PDE inhibitors to optimize lung exposure and minimize systemic circulation and the associated adverse effects. An inhaled formulation may deliver high concentrations directly to local inflammatory cells, ASM, and airway epithelium, potentially enabling efficacy at levels far lower than those required to produce systemic side effects. A second layer of specificity is the inhibitors’ selective for isoenzymes and even splice variants. As noted earlier, PDE4D appears to play a critical role in β2-adrenoceptor signaling and ASM contractility, whereas other variants may regulate distinct functions (Niimi et al. 2012). A drug with high selectivity for a splice variant of importance to the lung would, in theory, possess an improved side effect profile. Third, there is a therapeutic opportunity in the concept of dual or pan-PDE inhibition in a single molecule, such as a PDE3/4 or PDE4/7 inhibitor. As shown preclinically with RPL 554, lower doses of each inhibiting component may be used to target multiple pathways simultaneously, reducing the side effects of high-dose inhibition of a single PDE while achieving synergistic anti-inflammatory and bronchodilatory effects (Wójcik-Pszczoła et al. 2023). Such a strategy underscores the interconnectivity of PDE-mediated signaling networks in the lung. This analysis points to unrecognized potential benefits of PDE inhibition on airway mucosal defense and remodeling, beyond bronchodilation and direct immune cell suppression. By analogy with the effects of roflumilast on ciliary beat frequency and CFTR activity, these drugs can be expected to improve MCC. Such an effect would be highly beneficial in asthma phenotypes characterized by airway occlusion due to excessive mucus production. Additionally, modulation of cAMP and cGMP can affect key processes in airway remodeling: ASM proliferation, fibroblast activation, and extracellular matrix deposition. Long-term PDE inhibition may reduce these structural changes, particularly in severe or corticosteroid-resistant asthma; however, this has not been studied in detail in early clinical investigations (Niimi et al. 2012; Wójcik-Pszczoła et al. 2023).

The response to PDE inhibitors may not be consistent, given that the pattern of expression of PDE isoenzymes can vary significantly between individuals, and even in severe asthmatics, for example, defective NO-sGC-cGMP signaling (Ghosh et al. 2021). Biomarkers defining PDE-driven diseases in specific patients, such as a dominant “PDE4-high” inflammatory profile or “PDE3/5-high” ASM dysregulation, could enable targeted patient selection for clinical trials and potential treatment (Ghosh et al. 2021). This approach will replace the “one-size-fits-all” testing that could have hidden benefits in specific subpopulations in previous studies. Additionally, there is a short-term possibility of combining PDE inhibitors with existing therapies. The PDE3/4 inhibitors have pharmacologically potent synergistic interactions with both β2-agonists and muscarinic antagonists *in vitro* and *in vivo* (Mokry & Mokra 2018). Notably, a PDE inhibitor might allow a reduction in β2-agonist dosage and assuage concerns about tolerance. Additionally, PDE inhibitors may provide an alternative anti-inflammatory modality in corticosteroid-resistant asthma, either restoring responsiveness or offering additive control (Hakim et al. 2012). Furthermore, inhaled pan-PDE inhibitors reduced the OVA-induced airway inflammatory cell infiltration, eosinophil recruitment, Th2 cytokine level in bronchoalveolar lavage fluid, as well as both total and ovalbumen-specific IgE levels in plasma (Wójcik-Pszczoła et al. 2023). Thus, pan-PDE inhibitors administered by inhalation are dual-acting agents targeting both airway inflammation and remodeling in challenged allergic asthma and may represent promising anti-asthmatic drug candidates. Moreover, selective PDE4 inhibitors, such as roflumilast or cilomilast, and pan-PDE inhibitors might provide better inhibition of transforming growth factor β_1_ (TGF-β_1_)-induced ASMC remodeling. The recently synthesized 7,8-disubstituted purine-2,6-dione derivatives, in addition to being pan-selective PDE inhibitors, can interact with transient receptor potential ankyrin 1 (TRPA1) ion channels, which are implicated in the pathogenesis of asthma (Chłoń-Rzepa et al. 2018a, b; Chłoń-Rzepa et al. 2018a, b). It has been illustrated that 832 (a pan-PDE inhibitor), 869 (a TRPA1 modulator), and 145 (a pan-PDE inhibitor and a TRPA1 modulator) can limit profibrotic responses of lung fibroblasts (Wójcik-Pszczoła et al. 2023).

In conclusion, the targeting of PDEs in asthma is a logically effective but clinically unrealized approach thus far. To move forward, lessons have to be learned from past mistakes. Success will likely require innovative medication delivery, such as inhalation; complex molecular design, such as dual inhibitors or splice-variant-selective agents; and precision-guided administration based on biomarkers. By embracing, instead of trying to overcome, the intricacy of the PDE signaling network in the lung, future treatments may fully realize this pathway’s potential and thereby surmount the limitations imposed by broad systemic PDE4 suppression. This would finally achieve the initial goal of individualized, more effective treatment for this heterogeneous disease and provide new hope for asthma phenotypes poorly controlled by current therapies, particularly those characterized by combined bronchoconstriction and inflammation, with impaired mucosal clearance and remodeling.

Taken together, severe and inadequately controlled asthma remains a clinical challenge. For this reason, clinical trials and preclinical experimental studies on novel agents as add-on therapies continue to emerge. Novel PDE inhibitors exhibiting fewer adverse events may have a role as add-on therapies in asthma treatment in the future. More clinical trials are necessary to demonstrate their efficacy and evaluate their safety profile before regulatory bodies approve.

## Limitations and future directions

The present review had several limitations, including the lack of evaluation of the long-term efficacy and safety of PDE inhibitors, due to limited longitudinal and epidemiological studies of selective PDE inhibitors in patients with asthma. In addition, the cellular and molecular mechanisms of PDE inhibitors were estimated in preclinical studies, which have not been fully translated into clinical settings. Hence, further studies are recommended in this regard.

Although substantial progress has been made in defining the roles of PDEs in airway biology, current evidence remains limited by several factors, including the predominance of preclinical studies, inconsistencies across animal models, and the lack of isoform-specific clinical biomarkers that can reliably predict therapeutic responsiveness. The clinical development of systemic PDE4 inhibitors has been further constrained by dose-limiting gastrointestinal and neurological toxicities, making it difficult to delineate their true therapeutic potential in asthma. Additionally, most prior trials did not account for the marked heterogeneity of asthma genotypes or the variable expression of PDE isoforms across airway smooth muscle, epithelium, and immune cells, thereby obscuring potential benefits within specific patient subgroups. Future research must therefore prioritize the development of inhaled PDE inhibitors that maximize pulmonary exposure while minimizing systemic adverse effects, as well as next-generation isoform- and splice-variant–selective agents capable of precisely modulating compartmentalized cAMP and cGMP signaling. The strong preclinical results observed with dual PDE3/4 inhibition also warrant rigorous evaluation in well-designed translational studies, particularly in combination with β2-agonists or muscarinic antagonists to restore bronchodilator responsiveness in severe or corticosteroid-resistant asthma. Most importantly, integrating molecular genotyping, multi-omics profiling, and biomarker-driven patient selection into future clinical trials will be essential to overcoming past translational failures and identifying asthma phenotypes most likely to benefit from PDE-targeted therapy. By addressing these limitations and advancing toward precision medicine–based strategies, the therapeutic promise of PDE modulation may finally be realized for refractory asthma populations.

## Conclusion

The key role of PDEs in regulating bronchoconstriction, inflammation, and MCC function could be a logical approach for treating asthma. However, the systemic adverse effects that have arisen from broad-spectrum PDE4 inhibitors highlight the difficulty of finding a workable treatment window clinically. Hence, a paradigm shift toward precision medicine is needed to overcome this translational challenge. Future studies should focus on three specific directions: (1) Development of PDE inhibitor inhalation formulations that enhance lung-specific delivery while minimizing systemic exposure. (2) Development of next-generation PDE inhibitors with splice-variant selectivity, e.g., toward PDE4D or with dual action against PDE3/4 that achieves increased tolerance and efficacy through synergistic mechanisms. (3) Elucidation of asthma genotypes defined by specific biomarkers, such as the “PDE-high” or defective cGMP signaling phenotype, that enable focused stratification of patients in treatment trials. A combination of these innovative approaches-local delivery, improved inhibitor design, and biomarker-based therapy-may enable the development of PDE modulation as an effective therapeutic strategy in the management of asthma.

## Data Availability

All source data for this work (or generated in this study) are available upon reasonable request.
